# Off-pump *versus* On-pump Coronary Artery Bypass Grafting in Frail Patients: Study Protocol for the FRAGILE Multicenter Randomized Controlled Trial

**DOI:** 10.21470/1678-9741-2017-0196

**Published:** 2017

**Authors:** Omar Asdrúbal Vilca Mejía, Michel Pompeu Barros Oliveira Sá, Maurilio Onofre Deininger, Luís Roberto Palma Dallan, Rodrigo Coelho Segalote, Marco Antonio Praça de Oliveira, Fernando Antibas Atik, Magaly Arrais dos Santos, Pedro Gabriel Melo de Barros e Silva, Rodrigo Mussi Milani, Alexandre Ciappina Hueb, Rosangela Monteiro, Ricardo Carvalho Lima, Luiz Augusto Ferreira Lisboa, Luís Alberto Oliveira Dallan, John Puskas, Fabio Biscegli Jatene

**Affiliations:** 1 Instituto do Coração do Hospital das Clínicas da Faculdade de Medicina da Universidade de São Paulo (InCor-HCFMUSP), São Paulo, SP, Brazil.; 2 Division of Cardiovascular Surgery, Pronto-Socorro Cardiológico de Pernambuco (PROCAPE), Recife, PE, Brazil.; 3 Universidade de Pernambuco (UPE), Recife, PE, Brazil.; 4 Hospital Alberto Urquiza Wanderley - Unimed, João Pessoa, PB, Brazil.; 5 Instituto Nacional de Cardiologia (INC), Rio de Janeiro, RJ, Brazil.; 6 Hospital Beneficência Portuguesa de São Paulo, São Paulo, SP, Brazil.; 7 Instituto de Cardiologia do Distrito Federal, Brasília, DF, Brazil.; 8 Instituto Dante Pazzanese de Cardiologia, São Paulo, SP, Brazil.; 9 Total Cor Hospital - São Paulo, SP, Brazil.; 10 Pontificia Universidade Católica do Paraná (PUCPR), Curitiba, PR, Brazil.; 11 Hospital das Clinicas Samuel Libânio, Pouso Alegre, Minas Gerais, Brazil.; 12 Mount Sinai Heart at Mount Sinai Saint Luke's New York, NY, USA.

**Keywords:** Randomized Controlled Trial, Frail Elderly, Coronary Artery Bypass, Off-Pump

## Abstract

**Introduction:**

Advances in modern medicine have led to people living longer and healthier lives. Frailty is an emerging concept in medicine yet to be explored as a risk factor in cardiac surgery. When it comes to CABG surgery, randomized controlled clinical trials have primarily focused on low-risk (ROOBY, CORONARY), elevated-risk (GOPCABE) or high-risk patients (BBS), but not on frail patients. Therefore, we believe that off-pump CABG could be an important technique in patients with limited functional capacity to respond to surgical stress. In this study, the authors introduce the new national, multicenter, randomized, controlled trial "FRAGILE", to be developed in the main cardiac surgery centers of Brazil, to clarify the potential benefit of off-pump CABG in frail patients.

**Methods:**

FRAGILE is a two-arm, parallel-group, multicentre, individually randomized (1:1) controlled trial which will enroll 630 patients with blinded outcome assessment (at 30 days, 6 months, 1 year, 2 years and 3 years), which aims to compare adverse cardiac and cerebrovascular events after off-pump *versus* on-pump CABG in pre-frail and frail patients. Primary outcomes will be all-cause mortality, acute myocardial infarction, cardiac arrest with successful resuscitation, low cardiac output syndrome/cardiogenic shock, stroke, and coronary reintervention. Secondary outcomes will be major adverse cardiac and cerebrovascular events, operative time, mechanical ventilation time, hyperdynamic shock, new onset of atrial fibrillation, renal replacement therapy, reoperation for bleeding, pneumonia, length of stay in intensive care unit, length of stay in hospital, number of units of blood transfused, graft patency, rate of complete revascularization, neurobehavioral outcomes after cardiac surgery, quality of life after cardiac surgery and costs.

**Discussion:**

FRAGILE trial will determine whether off-pump CABG is superior to conventional on-pump CABG in the surgical treatment of pre-frail and frail patients.

**Trial registration:**

ClinicalTrials.gov, ID: NCT02338947. Registered on August 29^th^ 2014; last updated on March 21^st^ 2016.

**Table:** 

Abbreviations, acronyms & symbols				
ARDS	= Acute respiratory distress syndrome		OR	= Odds ratio
BBS	= Best bypass surgery		PCI	= Percutaneous coronary intervention
CABG	= Coronary artery bypass grafting		PROM	= Predicted risk of mortality or major morbidity
CI	= Confidence interval		RCTs	= Randomized controlled trials
CORONARY	= CABG Off- or On-Pump Revascularization Study		ROOBY	= Randomized on/off bypass
HR	= Hazards ratio		STS	= Society of Thoracic Surgeons

## INTRODUCTION

One of the most controversial areas of cardiac surgery has been whether off-pump coronary artery bypass grafting (OPCAB) surgery is superior to conventional on-pump coronary artery bypass grafting (CABG) surgery. There is an ongoing debate about the benefits and disadvantages of OPCAB. Initial trials have shown that off-pump CABG is feasible in selected low-risk patients and offers results similar to those of CABG performed with the conventional on-pump technique (on-pump CABG)^[1,2]^. In institutions with experience in off-pump CABG, the rates of major adverse events and of complete revascularization and graft patency have been similar to those with on-pump CABG^[3]^. These positive results have been called into question by reports of inferior graft patency and higher rates of repeat target-vessel revascularization associated with off-pump CABG^[4,5]^.

The Randomized On/Off Bypass (ROOBY) trial^[6]^ showed that, among low-risk patients, the rate of death or major adverse events at 30 days after surgery was similar with off-pump and on-pump CABG, but off-pump CABG was associated with a higher rate of incomplete revascularization at 1 year.

Short-term mortality and morbidity after off-pump and on-pump CABG were similar in a recent trial involving 4752 patients with a mixed operative-risk profile (the CABG Off- or On-Pump Revascularization Study [CORONARY])^[7]^.

The German Off-Pump Coronary Artery Bypass Grafting in Elderly Patients (GOPCABE)^[8]^ study focused exclusively on patients 75 years of age or older. However, this trial would not elucidate the potential benefit of off-pump CABG in high-risk patients because this specific group of German patients were moderate risk patients.

The results of the Best Bypass Surgery (BBS) trial^[9]^, performed on 341 high-risk patients (European System for Cardiac Operative Risk Evaluation "EuroSCORE">5) undergoing on-pump CABG or off-pump CABG, reported no significant differences in the composite of adverse cardiac and cerebrovascular events or in any of the following outcomes: all-cause mortality, acute myocardial infarction, cardiac arrest, low cardiac output/cardiogenic shock, stroke, and coronary reintervention.

However, in our opinion, the definition of high-risk patient should be interpreted carefully, since the EuroSCORE identifies patients based on 18 independent variables, many of which were not considered in the study. Risk factors such as previous cardiac surgery, critical preoperative state, emergency operation, and poor left ventricular dysfunction were excluded. Furthermore, active endocarditis, pulmonary hypertension, other than isolated CABG, surgery on the thoracic aorta, and postinfarction septal rupture were not considered due to the nature of the study. Chronic pulmonary disease and neurological dysfunction were not defined according to EuroSCORE, and there is no information on patients with unstable angina.

In the real world, with more than 1500 patients, we showed lower mortality among patients who underwent off-pump CABG^[10]^. So, after a certain cut-off EuroSCORE > 4.5 or 2000 Bernstein-Parsonnet score >17.75, off-pump CABG significantly reduces death rates. Indeed, numerous large retrospective studies and meta-analyses have shown significant short-term improvements after OPCAB and comparable long-term outcomes. A recent risk-adjusted analysis of the national Society of Thoracic Surgeons (STS) database assessing 876,081 patients demonstrated a significant reduction in death and stroke (11% and 34% reduction, respectively) after OPCAB, seen in both low- and high-volume centers^[11]^. After that, it is important to reconsider the best approach for patients with higher surgical risk for CABG.

Clearly, a base is being built with strong scientific evidence that this is the group that experiences the most benefit from off-pump CABG. This evidence allows not using cardiopulmonary bypass to be the main surgical approach, and complete revascularization and greater use of arterial grafts in patients with high surgical risk as the second plan^[12]^.

Actually, randomized controlled trials (RCTs) have failed to demonstrate a significant mortality benefit for OPCAB^[13]^. However, the available RCTs were underpowered to detect significant differences between the groups and suffered from high selection and exclusion biases. Even more important is the fact that the available RCTs so far have primarily focused only on low-risk (ROOBY, CORONARY), elevated-risk (GOPCABE) or highrisk patients (BBS), but not on frail patients in whom the benefits of OPCAB should be well defined. Moreover, the conversion rates (12.4% in ROOBY, 7.9% in CORONARY, and 5% in GOPCABE) may suggest that some of the participating surgeons were inexperienced because expert centers report conversion rates between 2% and 4%^[14]^, which significantly affects long-term outcomes.

We believe that avoiding cardiopulmonary bypass should be viewed primarily as a step toward avoiding aortic manipulation. Despite the long-term benefits of surgery, some patients may choose percutaneous coronary intervention (PCI) for the treatment of complex multivessel disease to avoid the morbidity associated with CABG, of which stroke is the most feared. Expert OPCAB surgeons can offer equivalent durability of graft patency as in CABG, with a lower rate of stroke if aortic manipulation is avoided.

Authors make mistakes in pointing out that on-pump CABG should be able to be performed under all circumstances, on all patients, at all institutions, regardless of their cardiac volume^[14]^. Thus, for example, owing to this rather conflicting evidence, either PCI or CABG surgery could be offered to patients with chronic kidney disease, depending on the complexity of coronary disease and comorbidities. If PCI is recommended, appropriate measures should be employed to prevent contrast-induced worsening of renal function. If CABG surgery is the preferred revascularization strategy, the off-pump technique might reduce the risk of acute kidney injury^[15]^.

On the other hand, cardiac scores, including EuroSCORE and STS, have been developed to predict the risk of adverse outcomes following surgery. Frailty, an independent predictor of mortality and complications, is not included in these risk algorithms. Emerging evidence suggests that frailty is a better marker of biological age and more important than chronological age^[16,17]^. Afilalo et al.^[18]^ determined that patients with slow preoperative gait speed (≥6 s to walk 5 m) had a 2 to 3 fold increased risk of mortality and major morbidity for any given level of STS-Predicted Risk of Mortality or Major Morbidity (PROM) compared with normal speed. Gait speed added to STS-PROM marginally increased model performance from 0.70 (0.60-0.80) to 0.74 (0.64-0.84). Forty-three percent of patients died or sustained a major complication assessed as high STS-PROM risk (≥15%) together with slow gait speed, compared with 21.7% low STS risk with slow gait and 18.9% high STS risk with normal gait. Afilalo et al.^[19]^ subsequently evaluated the prognostic value of various frailty, disability and cardiac risk scores to identify the optimal combination to predict adverse outcome. Patients with slow gait speed and ≥3 impairments on the Nagi disability scale predicted in-hospital morbidity and mortality above that of the Parsonnet cardiac risk score (AUC 0.76 *vs.* 0.72 with Parsonnet score alone). Lee et al.^[20]^ performed a retrospective review of a large cardiac registry, comparing outcomes between non-frail and frail individuals (coded as having deficiencies in the activities of daily living, need for walking aids or diagnosis of dementia). Frailty was an independent risk factor for in-hospital mortality (risk-adjusted odds ratio [OR] 1.8; 95% confidence interval [CI] 1.1-3.0; *P*=0.03) and mortality at 2 years (risk-adjusted hazards ratio [HR] 1.5, 95% CI 1.1-2.2; *P*=0.01).The benefits of CABG without cardiopulmonary bypass in pre-frail and frail patients are still undetermined.

In conclusion, frailty is defined as a geriatric syndrome of impaired resiliency to stressors (such as cardiac surgery) that has been delineated recently in the cardiovascular literature. However, the benefits of CABG without cardiopulmonary bypass in these patients are still undetermined. We believe OPCAB remains an important technique for the improvement of coronary surgery. The biggest question right now is whether pre-frail and frail patients will benefit more from off-pump or on-pump CABG.

The aim of this paper was to describe the FRAGILE trial protocol which intends to clarify the potential benefit of off-pump CABG in pre-frail and frail patients; we will conduct a national multicenter RCT comparing off-pump *versus* on-pump CABG in frail patients.

## METHODS

### Study Design

The FRAGILE study will be a national multicenter RCT to be conducted in 10 Brazilian institutions. The study is already approved by a certified ethics committee. The study sponsor must be the Brazilian Society of Cardiovascular Surgery and Zerbini Foundation. Funding will be provided by an unrestricted grant from São Paulo Research Foundation, which otherwise will not have any role in the conduct of the study nor in the analysis nor in the reporting of data. There will be confidentiality agreement regarding data use. This RCT will be monitored by an independent data and safety monitoring board. All the authors will be provided revisions and comments. All the authors will be testifying for the accuracy and completeness of the report, as well as for the fidelity of the report to the study protocol.

### Study Population

The inclusion criteria will be: participants aged ≥65 years with indication of myocardial revascularization with ≥2 criteria of frailty by Fried Frailty Criteria^[16]^, not eligible for angioplasty treatment by the heart team approach and those suitable to undergo either off-pump or on-pump CABG.

There is a dose-response relationship, in relation to the number of fragility criteria and patient outcomes. The categorization of the fragility variable was performed after the studies showed that the risk increases gradually over 3 categories (0-1, 2-3, 4-5), with risk of similar events within each category. Thus, patients who had 2 or 3 criteria were considered as pre-frail, and patients with 4 or 5 criteria were considered as frail^[21]^.

The exclusion criteria will be: patients with indication of another procedure in addition to CABG; patients who underwent emergency operation (within 24 hours after hospital admission); patients who underwent previous cardiac surgery, even with other approaches than median sternotomy; patients who do not have free, prior and informed consent to participate in this study.

The baseline characteristics of potentially eligible but excluded patients will be recorded in a screening log. All patients will be provided written informed consent.

### Participating Centers

1. Instituto de Cardiologia do Distrito Federal
2. Instituto do Coração da Faculdade de Medicina da Universidade de São Paulo
3. Pronto-Socorro Cardiológico de Pernambuco (PROCAPE)
4. Instituto Dante Pazzanese de Cardiologia
5. Beneficência Portuguesa de São Paulo
6. Instituto Nacional de Cardiologia
7. Pontifícia Universidade Católica do Paraná
8. Hospital das Clínicas Samuel Libânio de Pouso Alegre
9. Hospital Alberto Urquiza Wanderley de João Pessoa
10. Total Cor Hospital

### Randomization and Treatment

Eligible patients will be randomly assigned to off-pump CABG or on-pump CABG. Randomization will be performed after the baseline data, including information about the target vessels have been entered into a central, Internet-based, password-protected database with the use of a template.

Treatment assignments will be performed in a blinded manner according to a blocked randomization scheme with a block size of ten, stratified according to the participating center. Off-pump CABG must be routinely performed at all participating centers before the trial is initiated. Participating centers should nominate individual study surgeons for each surgical technique.

Study surgeons will be required to be established experts in the performance of either off-pump or on-pump CABG. The average number of CABG surgeries performed before the study will be between 50-100 off-pump surgeries for the off-pump CABG surgeons and 250 on-pump surgeries for the on-pump CABG surgeons. It will not be mandatory for the same surgeon who operates the off-pump CABG cases to also operate the on-pump CABG cases, but always respecting the established protocols for each surgical technique.

### Surgical Technique

Surgical access to the heart will be gained through a median sternotomy in all of the patients. In order to reduce the risk of bleeding and transfusions an absorbable hemostat is used to sternal bone marrow and recovery of red blood cells in all patients. Off-pump surgery will be performed with the use of heart stabilizers. Patients will be heparinized with 250 IU/kg intravenously to achieve activated clotting time >200s. The proximal anastomosis will be performed according to protocol to be discussed with our advisor. The distal anastomosis will be constructed with the help of mechanical stabilizers and cardiac positioner. Intracoronary shunts will be used routinely. On-pump surgery will be performed in normothermia, with the use of aortic cross-clamping and cold cardioplegic arrest. Patients will be heparinized with 500 IU/kg to achieve an activated clotting time >480 s. Heparin will be neutralized with 1 mg protamine sulfate per 5000 IU given. During the trial, there will be no changes in the 2 surgical techniques that followed a pre-established protocol.

### Data Storage, Follow-Up and Outcome Measures

All variables from the study will be stored online through a REDCap Web Platform (http://www.project-redcap.org/).The REDCap is a secure web application for building and managing online surveys and databases. REDCap can be used to collect virtually any type of data, it is specifically geared to support data capture for research studies. This will be done through national healthcare card, address and telephone numbers. On the basis of the registers, it will be collected copies of hospital records and death certificates during the follow-up period. The neurocognitive tests will be planned in accordance with the statement of consensus of assessment of neurobehavioral outcomes after cardiac surgery^[22]^.

We will assess health-related quality of life using the World Health Organization quality of life questionnaire^[23]^. Neurocognitive and quality of life tests will be carried out at baseline and at 6 months postoperatively. No follow-up visits will be planned; however, patients will be routinely seen by the referring cardiologist 1 and 6 months after the operation. Hospital records and death certificates will be blinded for the allocated treatment and forwarded to 2 randomly selected members of the adjudication committee, who will assess whether each of the pre-specified outcomes had occurred. In case of disagreement, the 2 assessments together with a copy of the record of the event will be sent to a third member, who will have to select the most likely assessment.

### The Primary Endpoint

It will comprise mortality rates and complications (allcause death, acute myocardial infarction, stroke, renal failure, acute respiratory distress syndrome and bleeding reoperation) occurring within 30 days of surgery. Hospital mortality is defined as death occurring within 30 days after surgery. Myocardial infarction is defined as the appearance of a new Q wave on the electrocardiogram with an increase of CKMB> 100 IU/L and/or> 10% of the total CK level and/or new wall movement abnormalities, with the exception of the septum, documented on the echocardiogram. Neurological complications are defined as stroke (neurological deficit> 24 hours with positive findings on computed tomography) or transient ischemic attack (neurological deficit <24 hours with positive findings on computed tomography). Renal insufficiency is defined as an increase in plasma creatinine> 2 associated with a urinary output <0.5 mL kg/h/in 12 hours. Acute respiratory distress syndrome (ARDS) is defined as the presence of tachypnea (respiratory rate> 30 breaths/min), bilateral pulmonary infiltrate on chest radiography, severe hypoxemia (arterial oxygen partial blood pressure/inspired oxygen fraction <200), the need for positive pressure end-expiratory flow> 5 cmH2O, no evidence of left ventricular failure (wedge pulmonary capillary pressure <18 mmHg), and no pathological features to explain these findings. Reoperation for bleeding is defined as the need to chest reopening in the presence of> 500 mL of blood by the chest tube within the first hour, >400 mL within the second hour, >300 mL within the third hour, or total bleeding >1000 mL within the fourth hour.

### Secondary Outcome Measures

- Major adverse cardiac and cerebrovascular events after OPCAB and CABG in pre-frail and frail patients (1 year)
- Major adverse cardiac and cerebrovascular events after OPCAB and CABG in pre-frail and frail patients (2 years)
- Operative time (180 days)
- Mechanical ventilation time (180 days)
- Hyperdynamic shock (180 days)
- New onset of atrial fibrillation (180 days)
- Need for pacing >24 hours (180 days)
- Renal replacement therapy (180 days)
- Reoperation for bleeding (180 days)
- Pneumonia (180 days)
- Length of stay in intensive care unit (180 days)
- Length of stay in hospital (180 days)
- Transfusion requirement (180 days)
- Graft patency assessed by coronary computed tomography (1 year)
- Correlation between clinical and angiographic scores to prognosis (1 year)
- Recurrence of angina (1 year)

### Other Endpoints

- Neurobehavioral outcomes after cardiac surgery (180 days)
- Quality of life after cardiac surgery (180 days)
- Cost (180 days)

### Statistical Analysis

The sample size of the FRAGILE trial was based on the ability to detect a 40% reduction in the primary outcome in the off-pump group compared with the on-pump group, assuming an event proportion after on-pump CABG of 20.8% and accepting a risk of type I and II error of 5% and 20%, respectively, and a crossover rate of 5%^[24]^. Consequently, at least 630 patients will be enrolled.

All of the data will be analyzed according to intention-to-treat (*i.e.*, based on treatment allocation). Therefore, we will report treatment-received analyses according to the intervention actually received. Baseline characteristics and operative characteristics will be compared with the use of chi-square test, t-test, or Kruskal-Wallis test, as appropriate. Dichotomous data will be presented as numbers and percentages. Continuous data will be presented as mean and standard deviation or median and interquartile range. The continuity corrected chi-square test will be used for comparison of the 30-day endpoint. Mantel-Haenszel chi-square tests will be used to adjust for study-center effects. Treatment effects at 30 days will be expressed as OR and 95% CI. For the 12-month endpoint, Kaplan-Meier curves will be constructed, and the study groups will be compared with use of the log-rank test. HR and 95% CI derived from the Cox proportional hazards model will be provided for the composite outcome and individual components. All statistical analyses will be performed with the use of the R-3.1.2 language for handling and storage of data and performing of statistical calculations.

## DISCUSSION

The elderly represent the fastest growing group of patients referred for cardiac surgery, with the proportion of patients aged 75 years or older steadily rising from 16% in 1990 to 25% in most recent estimates^[25]^. Advanced age is frequently accompanied by a larger burden of comorbid conditions and greater illness severity. In the setting of cardiac surgery, elderly patients are more likely to have extensive coronary artery disease and concomitant valvular disease and are more likely to require urgent or emergent surgery^[26]^.

Nevertheless, elderly patients have consistently been shown to derive sizeable benefits from cardiac surgery^[27]^. This risk-benefit dichotomy renders the process of selecting appropriate elderly patients particularly challenging for the cardiac surgeon.

Frailty is defined as a geriatric syndrome of impaired resiliency to stressors that has been delineated recently in the cardiovascular literature^[28]^. In several observational studies and registry data in which detailed statistical analyses were carried out, mortality and morbidity have been found to be significantly reduced after off- pump CABG compared with on-pump CABG in high-risk patients (*e.g.*, patients with advanced age or cardiac and systemic comorbidity)^[8,12-14]^.

The results of BBS randomized trial are in contrast to these findings. Overall 30-day mortality in this trial was 4.4%, which was less than the predicted mortality rate of 7% according to the EuroSCORE but in agreement with the predicted 3.1% according to the STS risk score. This study shows that the risk patients were overestimated by the EuroSCORE and this sample will be composed of low and moderate risk patients by the STS risk score^[9]^.

In the GOPCABE trial, there was no significant difference between off-pump CABG and on-pump CABG performed in elderly patients with respect to the composite endpoint of death, stroke, myocardial infarction, repeat revascularization, or new renal replacement therapy after surgery. With a mean age of 78 years and a predicted in-hospital mortality of 3.8%, the study cohort does not represent a population with a high operative risk. The results of previous studies are consistent with regard to high-risk patients^[16-18]^.Therefore, this trial does not support the assumption that off-pump CABG cannot improve the early outcome in high-risk patients.

In contrast to the ROOBY trial^[11]^, the GOPCABE study did not show a significant difference in survival or major adverse events at 1 year after surgery. This absence of difference may be a consequence of the requirement for substantially greater experience with off-pump CABG in the GOPCABE trial than in the ROOBY trial.

With the growth of minimally invasive and transcatheter cardiac interventions, the expansion of risk prediction beyond traditional risk factors and risk scores has become a high priority. Clinicians may use this integrative approach of combining risk scores including frailty and disability to better characterize elderly patients referred for cardiac interventions and identify those who are vulnerable and at increased risk. A multidisciplinary team involving cardiac surgery, cardiology, geriatric medicine, physiotherapy, and occupational therapy may be well suited to address these diverse elements which contribute to postoperative risk^[19]^.

The CORONARY trial falls short of delivering a knockout victory for the off-pump technique. There were no significant differences in the rate of the composite primary outcome of death, stroke, non-fatal myocardial infarction or new renal failure at 30 days between off-pump and on-pump CABG. However, off-pump CABG was associated with fewer transfusions, reoperations for bleeding, acute kidney injury, and respiratory infections or failure. Thus, the off-pump technique may be best in certain subgroups of patients and we must choose the procedure that is best for them^[29]^. We hypothesized that the frailty status would contribute to the risk in patients undergoing on-pump CABG and that off-pump CABG could be advantageous for frail patients.

### Limitations

Some limitations of our trial are to be noted. Firstly, the assessment of graft patency will not be performed by coronary catheterization (test which indicated surgery). In contrast, coronary computed tomography angiogram will be performed in all patients for this purpose. Secondly, the events will not be adjudicated by a blinded adjudication committee. All data will be provided by the local investigators according to the protocol definitions.

**Table:** 

Authors' roles & responsibilities	
OAVM	Conception and design of the work; drafting the work and revising it critically; final approval of the version to be published
MPBOS	Conception and design of the work; drafting the work and revising it critically; final approval of the version to be published
MOD	Conception and design of the work; drafting the work and revising it critically; final approval of the version to be published
LRPD	Conception and design of the work; drafting the work and revising it critically; final approval of the version to be published
RCS	Conception and design of the work; drafting the work and revising it critically; final approval of the version to be published
MAPO	Conception and design of the work; drafting the work and revising it critically; final approval of the version to be published
FAA	Conception and design of the work; drafting the work and revising it critically; final approval of the version to be published
MAS	Conception and design of the work; drafting the work and revising it critically; final approval of the version to be published
PGMBS	Conception and design of the work; drafting the work and revising it critically; final approval of the version to be published
RMM	Conception and design of the work; drafting the work and revising it critically; final approval of the version to be published
ACH	Conception and design of the work; drafting the work and revising it critically; final approval of the version to be published
RM	Conception and design of the work; drafting the work and revising it critically; final approval of the version to be published
RCL	Conception and design of the work; drafting the work and revising it critically; final approval of the version to be published
LAFL	Conception and design of the work; drafting the work and revising it critically; final approval of the version to be published
LAOD	Conception and design of the work; drafting the work and revising it critically; final approval of the version to be published
JP	Conception and design of the work; drafting the work and revising it critically; final approval of the version to be published
FBJ	Conception and design of the work; drafting the work and revising it critically; final approval of the version to be published
